# Geriatrische Aspekte bei Diabetes mellitus (Update 2023)

**DOI:** 10.1007/s00508-022-02124-w

**Published:** 2023-04-20

**Authors:** Joakim Huber, Michael Smeikal, Christoph H. Saely, Harald Stingl, Martin Clodi, Monika Lechleitner, Peter Fasching

**Affiliations:** 1 Abteilung für Innere Medizin mit Akutgeriatrie und Palliativmedizin, Franziskus Spital, Standort Landstraße, Landstraßer Hauptstraße 4a, 1030 Wien, Österreich; 2 Abteilung für Innere Medizin mit allgemeiner Geriatrie und Palliativmedizin, Haus der Barmherzigkeit, Wien, Österreich; 3 Abteilung für Innere Medizin und Kardiologie/VIVIT-Institut, Landeskrankenhaus Feldkirch, Feldkirch, Österreich; 4 Interne Abteilung, Landesklinikum Melk, Melk, Österreich; 5 Interne Abteilung, Landeskrankenhaus Hochzirl – Natters, Hochzirl, Österreich; 6 5. Medizinische Abteilung mit Endokrinologie, Rheumatologie und Akutgeriatrie, Klinik Ottakring der Stadt Wien, Wien, Österreich; 99 ICMR—Institute for Cardiovascular and Metabolic Research, Johannes Kepler Universität Linz (JKU Linz), 4040 Linz, Österreich

**Keywords:** Geriatrische Aspekte, Diabetes, Therapie, Empfehlungen, Glukose, Schulung, Alter, Diagnose, Individualisierung, Funktionelle Abhängigkeit, Geriatric, Elderly, Diabetes, Therapy, Recommendations, Glucose, Educational training, Diagnosis

## Abstract

Es besteht eine hohe Prävalenz an Diabetes mellitus Typ 2 bei über 70-Jährigen in industrialisierten Ländern. Dieser Artikel enthält Empfehlungen für Diagnose, Prävention und Therapieziele in der Behandlung älterer Menschen mit Diabetes anhand der aktuellen Evidenzlage.

## Abstract (en)

There is a high prevalence of diabetes mellitus in the elderly population of industrial countries. The present article provides recommendations for the screening, prevention and treatment of elderly diabetic patients according to current scientific evidence.

## Demographie

Die Prävalenz an Diabetes mellitus Typ 2 liegt bei über 70-Jährigen in industrialisierten Ländern bei 20–25 %. Werden systematisch auch Formen des „Prädiabetes“ (gestörte Nüchternblutglukose; pathologische Glukosetoleranz) erfasst, steigt der Prozentsatz der von Glukosestoffwechselstörungen betroffenen älteren Personen auf annähernd 50 % [1, 2]. In der westlichen Welt ist die bis 2030 prognostizierte Steigerung der Diabetesinzidenz vor allem durch den demographischen Wandel bedingt. Der Anteil eines autoimmunbedingten Diabetes (LADA-Diabetes) ist mit weniger als 5 % bei über 70-Jährigen gering.

## Screening

Generell sind zur Diagnose eines Diabetes die Bestimmung der Nüchternblutglukose, der 2 h Glukose (nach oraler Gabe von 75 g im OGTT) oder des HbA1c als gleichwertig anzusehen [3, 4]. Durch ausschließliche Erfassung der Nüchternblutglukose wird bei über 70-Jährigen häufig eine postprandiale Hyperglykämie im Sinne eines manifesten Diabetes mellitus übersehen, da mit zunehmendem Alter eine progrediente β‑Zell-Dysfunktion vorliegt [5]. Ein oraler Glukosetoleranztest wird zwar zur Abklärung der Stoffwechselsituation empfohlen, ist aber bei älteren Menschen oft technisch nicht möglich. Ein HbA1c-Wert von ≥ 6,5 % entspricht einem Diabetes mellitus und kann für ein Screening herangezogen werden. Ein HbA1c-Wert zwischen 5,7–6,4 % geht mit einem erhöhtem Diabetesrisiko einher [3, 4]. Auch Personen mit Erstdiagnose Diabetes im höheren Alter entwickeln makro- und mikrovaskuläre Komplikationen und leiden unter einer höheren Morbidität und Mortalität als gleichaltrige Personen ohne Diabetes [6], wobei dieser Effekt erst nach mehrjähriger Diabetesdauer nachweisbar ist [7]. Die Gesamtmortalität und der kardiovaskuläre Tod waren in einer Auswertung schwedischer Registerdaten in allen Altersgruppen (von < 55 bis ≥ 75 Jahren) abhängig von der Höhe des HbA1c (von ≤ 6,9 % bis ≥ 9,7 %) gesteigert. Die Hazard Ratio für Ereignisse nahm jedoch mit zunehmenden Alter ab [8].

## Prävention

Laut prospektiver Diabetespräventionsstudien (DPP) vermindert Lebensstilmodifikation (Ernährungsumstellung, geringe Gewichtsreduktion, Steigerung der körperlichen Aktivität) auch bei Risikopersonen mit gestörter Glukosetoleranz über dem 60. Lebensjahr die Diabetesinzidenz [9]. Prospektive Daten für über 70-Jährige liegen dazu aber nicht vor. In einer longitudinalen Kohortenstudie mit einem männlichen Kollektiv und durchschnittlichem Alter von 70 Jahren konnte das Diabetesrisiko durch regelmäßige körperliche Aktivität deutlich gesenkt werden [10]. In einem nicht-diabetischen adipösen Kollektiv mit einem Alter von 65 Jahren und darüber konnte gezeigt werden, dass vor allem eine Kombination von Gewichtsreduktion mit Diät und regelmäßiger körperlicher Aktivität die körperlichen Funktionen verbesserte [11]. Regelmäßige körperliche Aktivität und Krafttraining (wenn nicht kontraindiziert) sind bis ins höchste Alter gesundheitsfördernd, aber aufgrund physischer Limitationen im Alter häufig im Alltag nicht umsetzbar [12].

## Ernährung

Generell gelten auch für ältere und betagte Patienten mit Diabetes mellitus die gleichen Ernährungsempfehlungen wie für jüngere (s. Leitlinie Ernährung). Auf die Problematik einer iatrogenen Mangelernährung bei über 70-Jährigen wird hingewiesen. Davon sind vor allem multimorbide und pflegebedürftige Menschen betroffen. Eine Ernährung die an den kulturellen Hintergrund, persönliche Ziele und Vorlieben angepasst wird, erhöht die Lebensqualität, die Zufriedenheit mit den Mahlzeiten und verbessert den Ernährungsstatus [13]. Eine einseitige strikte „Diabeteskost“ in Pflegeheimen ist somit obsolet. Die adäquate Deckung des Energiebedarfs und die Erhaltung einer bestmöglichen Lebensqualität sind in dieser Betreuungssituation als vorrangige Ziele zu sehen. Die tägliche Aufnahme an Energie wird mit ca. 30 Kcal pro kg Körpergewicht empfohlen. Je nach Ernährungszustand, körperlicher Aktivität, Stoffwechselsituation und Toleranz muss dieser Wert individuell angepasst werden [14]. Ältere Menschen benötigen aufgrund der anabolen Resistenz und dem erhöhten Risiko für die Entwicklung einer Sarkopenie einen erhöhten Proteinanteil (1–1,5 g/kg/KG pro Tag) vor allem wenn sie körperliches Training (Muskelkräftigung) durchführen und sofern keine höhergradige Niereninsuffizienz vorliegt [15]. Unterschreitet die tägliche Kalorienzufuhr 1500 Kcal, ist mittelfristig mit Defiziten an Vitaminen und Spurenelementen zu rechnen. Eine entsprechende Supplementation wird empfohlen. Besonders zu beachten ist eine adäquate Zufuhr von Vitamin D und Calcium (s. Leitlinie Ernährung).

## Der geriatrische Patient, Frailty und Sarkopenie

Der geriatrische Patient ist durch seine Multimorbidität charakterisiert und hat meistens aber nicht zwingend auch ein hohes biologisches Lebensalter. Die Behandlung von alten Menschen mit Diabetes ist komplex, da es sich um ein heterogenes Kollektiv handelt. Manche sind weitgehend gesund, erkranken an Diabetes im fortgeschrittenen Alter und entwickeln keine Komplikationen, andere wiederum leiden an unterschiedlichen chronischen Erkrankungen und entwickeln beträchtliche diabetes-assoziierte Komplikationen, kognitive und funktionelle Einschränkungen oder Frailty [16, 17]. Zur Definition der Behandlungsziele sollten beim geriatrischen Patienten geriatrische Syndrome – und damit funktionelle und kognitive Einschränkungen, eingeschränkte Mobilität, Sturzneigung, chronische Schmerzen, Harninkontinenz oder Depression – Berücksichtigung finden. Diese geriatrische Multimorbidität stellt für den betroffenen Menschen im Alltag eine große Beeinträchtigung und darüber hinaus ein wichtiges Risiko für verminderte Lebensqualität dar. Geriatrische Syndrome treten bei älteren Menschen mit Diabetes mellitus signifikant häufiger auf als bei Gleichaltrigen ohne Diabetes (in manchen Studien sogar doppelt so häufig!) [7, 18–20].

Frailty („Gebrechlichkeit“) ist ein wichtiges geriatrisches Syndrom, welches mit einem Verlust von Selbständigkeit, einer funktionellen Beeinträchtigung und Abhängigkeit, sowie einer verminderten Reserve und Widerstandskraft gegenüber Stressoren verbunden ist. Folgen sind eine erhöhte Rate an Stürzen, Pflegebedürftigkeit, Unterbringung in einer Pflegeinrichtung und erhöhte Mortalität. Die häufig verwendete Definition für Frailty nach Fried beschreibt einen physischen Phänotyp mit 5 Kriterien: ungewollter Gewichtsverlust (> 5 kg in 12 Monaten), muskuläre Schwäche (mit Handkraftmessung festgestellt), subjektiv empfundene Erschöpfung, langsamer Gang (Ganggeschwindigkeit < 0,8 m/s über mindestens 4 m), niedriges physisches Aktivitätsniveau. Frailty besteht wenn mindestens 3 Merkmale zutreffen [21, 22].

Sarkopenie ist durch einen Verlust an Muskelmasse und Muskelfunktion im Alter gekennzeichnet und kann durch Messung der Handkraft oder der Ganggeschwindigkeit erkannt werden [23]. Sarkopenie und Frailty nach Fried überlappen sich in diesen beiden Diagnosekriterien. Der Muskel spielt eine wichtige Rolle für die Steuerung des Glukosestoffwechsels. Bei älteren Menschen mit Diabetes besteht eine höhere Prävalenz für Sarkopenie, eine verminderte Muskelmasse, eine verminderte Muskelqualität, eine verminderte Muskelkraft, eine geringere Ganggeschwindigkeit und ein beschleunigter Muskelabbau verglichen mit Kontrollen ohne Diabetes [24, 25]. Es wird empfohlen bei älteren Menschen mit Diabetes ein Frailty/Sarkopenie Screening durchzuführen [22]. Für beide stehen praxistaugliche Fragebögen mit einem Aufwand von wenigen Minuten zur Verfügung (SARC‑F für Sarkopenie und FRAIL für Frailty) [26, 27]. Für die Praxis scheint die Diagnose nach den Europäischen Sarkopenie-Kriterien bzw. nach den Frailty-Kriterien nach Fried zu umfangreich und nur unter stationären Bedingungen umsetzbar [28]. Für ältere Betroffene mit Diabetes und Frailty wird neben einem HbA1c von 7,5–8 % ein altersangepasstes körperliches Trainingsprogram, das auch ein Krafttraining und eine ausreichende Aufnahme von Energie bzw. Protein als therapeutische Intervention beinhalten soll, empfohlen [29–32].

Der Behandler muss die Heterogenität des älteren Menschen bei der Wahl der individuellen Therapieziele berücksichtigen. Zur Therapieplanung können Patienten mit Diabetes im Alter in unterschiedliche Gruppen mit unterschiedlichem Ausmaß an funktioneller Abhängigkeit eingeteilt werden (Tab. 1 und 2) (übernommen von [28]).

**Table 1:** 

Funktionell unabhängig	Ältere Menschen mit Diabetes und gutem funktionellen Status. *(weitgehend selbstständig und robust)* Patienten mit wenig Komorbidität, allenfalls geringer kognitiver Einschränkung und guten Kompensationsmöglichkeiten
Funktionell leicht abhängig	Ältere Menschen mit Diabetes und eingeschränktem funktionellen Status. *(geringer Pflege- bzw.-Betreuungsbedarf und gebrechlich –„pre-frail“ oder „frail“)* Patienten mit Multimorbidität, funktionellen und kognitiven Einschränkungen sowie geriatrischen Syndromen
Funktionell stark abhängig	Ältere Menschen mit Diabetes und extrem eingeschränktem funktionellen Status oder terminal erkrankte Menschen. *(hochgradiger Pflege- und Betreuungsbedarf und/oder „End of Life“)* Patienten mit Multimorbidität, geriatrischen Syndromen, ausgeprägten funktionellen und kognitiven Einschränkungen und Vorliegen von Erkrankungen mit limitierter Lebensprognose, z. B. terminale Herz- Nieren- oder maligne Erkrankungen

**Table 2:** 

Patientengruppe	Begründung	HbA1c	Blutglukose vor den Mahlzeiten	Blutdruck	Lipide
*Funktionell unabhängige Patienten:* Wenig Begleiterkrankungen, kognitiv nicht eingeschränkt	Lebenserwartung > 15 Jahre Vorteile einer intensiven Therapie können erlebt werden	< 7,0–7,5 %	100–130 ^c^ mg/dl	< 140/90 ^a^ mm Hg [55]	Statin beginnen wenn nicht kontraindiziert oder nicht toleriert, zielwert-orientierter Ansatz nach [56, 57]
*Funktionell leicht abhängige Patienten:* Sehr alte oder multimorbide oder kognitiv leicht eingeschränkte Patienten	Lebenserwartung < 15 Jahre Vorteile einer intensiven Therapie können nicht erlebt werden. Erhöhtes Hypoglykämie- und Sturzrisiko	≤ 8,0 % ^b^	100 ^a^–150 mg/dl	< 140/90 mm Hg	Statin beginnen wenn nicht kontraindiziert oder nicht toleriert, zielwert–orientierter Ansatz [56]
*Funktionell stark abhängige Patienten:* Pflegeabhängige oder kognitiv stark eingeschränkte Patienten oder End of life	Begrenzte Lebenserwartung	< 8,5 % ^b^	100 ^c^–180 ^c^ mg/dl	< 150/90 mm Hg Individuelle Therapieentscheidung, die den Gesamtkontext des Patienten einbezieht (da keine Zielwertevidenz)	Wahrscheinlichkeit eines Nutzen durch eine Statintherapie abschätzen (eher bei Sekundär – als bei Primärprävention zu erwarten)

Die Diagnose eines Typ 2 Diabetes erhöht das Risiko eines kognitiven Abbaus in den Folgejahren [33] bzw. das Risiko für die Entwicklung einer Demenz [34]. Eine schlechte aber auch instabile glykämische Kontrolle ist mit einem kognitiven Abbau assoziiert [35, 36]. Das erhöhte Risiko einer nachlassenden kognitiven Leistung kann aber durch Zielwerterreichung von Risikofaktoren zusätzlich zum HbA1c schrittweise reduziert werden [37]. Das Vorliegen einer kognitiven Beeinträchtigung kann das Erreichen von Therapiezielen erschweren, da komplexere Therapieumsetzungen, Selbstmanagement, Ernährungsumstellung, Dosisanpassung einer Insulintherapie oder Glukosekontrollen für den Patienten nicht durchführbar sind. Es ist daher wichtig einfache Therapiestrategien für Patienten mit Demenz zu wählen. Das Risiko einer Depression ist bei Diabetes um das 2‑fache erhöht [38]. Diabetiker mit Depressionen wiesen ein erhöhtes Risiko für die Entwicklung einer Demenz auf bzw. hatten eine erhöhte Gesamtmortalität und kardiovaskuläre Mortalität [39–42]. Bei Erwachsenen mit 65 Jahren oder älter wird ein Screening zur Erkennung einer kognitiven Beeinträchtigung, Demenz und Depression bei Erstvorstellung und danach jährlich je nach Bedarf empfohlen [43]. Für das Screening der Kognition kann der Minimental State Examination (MMSE) [44], der Mini-Cog [45] oder der Montreal Cognitive Assessment Test (MOCA) [46] verwendet werden. Bei Auffälligkeiten sollte eine diagnostische Abklärung inklusive neuropsychologischer Begutachtung folgen [47].

## Therapieziele (Glukose)

Generell gelten für ältere Menschen mit Diabetes die gleichen Stoffwechselziele wie für jüngere, wenn diese unter entsprechender Lebensstilführung und medikamentöser Therapie sicher und mit adäquater Lebensqualität erreicht werden können (s. Leitlinie Antihyperglykämische Therapie bei Diabetes mellitus Typ 2). In einer retrospektiven Kohortenstudie bei über 60-Jährigen kam es zu einem Anstieg der Mortalität bei einem HbA1c-Wert von < 6 % und > 8 % [48]. Eine zu aggressive Therapie bei älteren Patienten mit fortgeschrittener Erkrankung und Komplikationen hat nicht zu einer gesundheitlichen Verbesserung geführt, sondern eher geschadet [49]. Je höher das Lebensalter bei Erstdiagnose eines Diabetes mellitus ist, desto geringer werden auch die Unterschiede im altersentsprechenden Morbiditäts- und Mortalitätsrisiko im Vergleich zu Menschen ohne Diabetes (gezeigt für über 70-Jährige) [50]. Demzufolge können im Alter, in Abhängigkeit von individuell zu beurteilender Multimorbidität mit mehreren gleichzeitig bestehenden chronischen Erkrankungen, bei eingeschränktem funktionellen Status, funktioneller Abhängigkeit, bei Demenz und damit einhergehender verkürzter Lebenserwartung auch höhere HbA1c-Zielwerte toleriert werden (Tab. 2).

Eine optimale Diabetestherapie älterer Patienten sollte einerseits darauf abzielen, die Entwicklung dieser geriatrischen Syndrome zu verhindern und andererseits bei Vorhandensein dieser Problematik adäquate ganzheitliche Betreuungskonzepte im interdisziplinären Kontext anzubieten. Gründe für eine Anhebung der individuell festgelegten Stoffwechselziele sind: hohes Risiko für Hypoglykämien laut Anamnese (da Sturzgefahr und verschlechterte Kognition), Pflegebedürftigkeit, Multimorbidität, fortgeschrittene Demenz, funktionelle Abhängigkeit sowie begrenzte Lebenserwartung aufgrund einer konsumierenden oder progredienten Grundkrankheit (Tab. 2).

Eine chronische Erhöhung der Nüchternglukosewerte über 180 mg/dl bzw. der postprandialen Werte über 300 mg/dl erfordert jedenfalls eine Therapieintensivierung bzw. Therapieumstellung (z. B. Beginn einer Insulintherapie), da mit manifester Glukosurie und entgleister Hyperglykämie Dehydrierung, Infektionen, eine Verschlechterung der Kognition und Kachexie verbunden sind [53, 58].

Da gerade ältere Patienten nur eingeschränkt oder gar nicht auf Hypoglykämien reagieren und die dabei auftretenden Symptome oft unspezifisch (Schwindel, Schwäche, Verwirrtheit, Stürze) sind [59], sollten diese unbedingt vermieden werden. Schwere und häufigere Hypoglykämien sind mit einem erhöhten Risiko einer Demenzentwicklung assoziiert [60]. Umgekehrt führt eine schlechte kognitive Leistung zu einem erhöhten Risiko für schwere Hypoglykämien [61].

## Therapieziele (Blutdruck)

Prinzipiell erscheinen im höheren Lebensalter der systolische Blutdruck und der Pulsdruck (Blutdruckamplitude) als entscheidende Risikofaktoren für kardiovaskuläre Komplikationen [62]. Prospektive Interventionsstudien, welche ausschließlich alte diabetische Patienten eingeschlossen haben, liegen derzeit nicht vor. Das mittlere Alter bei Einschluss der Patienten in der ACCORD-Studie betrug 62 Jahre. In der ACCORD-Studie brachte die Absenkung der Zielblutdrucks auf systolisch unter 120 mm Hg im Vergleich zu einem Zielblutdruck von systolisch unter 140 mm Hg keine Reduktion kardiovaskulärer Endpunkte [49]. Ausschließlich über 80-jährige Patienten wurden in der HYVET-Studie inkludiert, wobei der Anteil an Personen mit Diabetes mit ca. 7 % und der Anteil an kardiovaskulärer Vorerkrankung mit ca. 12 % sehr gering war und die Patienten funktionell unabhängig waren. Die Erreichung eines Zielblutdruckwertes von < 150/90 mm Hg war mit einer signifikanten Reduktion der Gesamtmortalität um 21 % und mit einer signifikanten Reduktion der kardiovaskulären Ereignisse um 34 % assoziiert [63]. Im Vergleich zur HYVET Studie wurden in der SPRINT Studie ältere Patienten mit erhöhter Vulnerabilität und reduzierter Ganggeschwindigkeit rekrutiert. Die eingeschlossene Population hatte ein erhöhtes kardiovaskuläres Risiko aber keinen Diabetes. Der Anteil der > 75-jährigen lag bei 28 %. Eine stärkere Blutdrucksenkung auf 124/62 mm Hg führte zu einer signifikanten Reduktion von kardiovaskulären Ereignissen, Herzinsuffizienz und Gesamtmortalität um > 30 % bei allen Endpunkten verglichen mit einer Standardbehandlung und einem durchschnittlichen Blutdruck von 135/67 mm Hg [64]. In einer Meta-Analyse konnte gezeigt werden, dass es durch eine medikamentöse blutdrucksenkende Therapie auch im höheren Alter zu einer Reduktion von kardiovaskulären Ereignissen kam. Es sollte daher keine Differenzierung bei den Blutdruckzielen aufgrund des Alters vorgenommen werden [65]. Die ESC Prevention Guidelines 2021 empfehlen in behandelten Patientin > 70 Jahre einen systolischen Blutdruck generell auf < 140 mm Hg und eine weitere Senkung auf bis zu 130 mm Hg. Der diastolische Blutdruck soll auf < 80 mm Hg gesenkt werden [57]. Die American Diabetes Association unterstützt diese Ziele und empfiehlt generell einen Blutdruck von < 140/90 mm Hg. Bei multimorbiden und funktionell stark abhängigen Patienten liegt das Ziel bei < 150/90 mm Hg [43].

Die empfohlene Auswahl an Antihypertensiva entspricht jener für jüngere Patienten und orientiert sich an Komorbiditäten, Verträglichkeit, Nebenwirkungen und Kontraindikationen [55, 66].

## Therapieziele (Lipide)

Generell gelten für den älteren Diabetiker die gleichen Lipidzielwerte wie für den jüngeren, wenn diese unter entsprechender Lebensstilführung und medikamentöser Therapie sicher erreicht werden können. Dies trifft insbesondere auf funktionell unabhängige, aktive und selbstständige Personen zu. Die Leitlinien der ESC und des EASD empfehlen einen zielwert-orientierten Therapieansatz [57, 67, 68], der sich nach dem individuellen Risiko richtet (je nach Risiko LDL < 100 mg/dl bzw. < 70 mg/dl oder < 55 mg/dl) (Tab. 2). In prospektiven Interventionsstudien waren die erzielten relativen Risikoreduktionen vergleichbar mit denen jüngerer PatientInnen, die absolute Risikoreduktion gemäß dem höheren Hintergrundrisiko sogar größer [69, 70]. Geriatrische Patienten im Alter > 80 Jahren profitieren von einer Statintherapie in der Sekundärprävention hinsichtlich kardiovaskulärer Ereignisse [68, 71]. Der Beginn mit einer Statintherapie in der Primärprevention kann für Ältere > 70 Jahre in Erwägung gezogen werden, wenn ein hohes oder sehr hohes Risiko besteht [57]. Bei ausgeprägter Multimorbidität, fortgeschrittener Demenz und stark reduzierter Lebenserwartung, ist die Indikation zur lipidsenkenden Therapie auf Basis des Behandlungszieles aus Sicht des Patienten individuell und kritisch abzuwägen.

## Orale Diabetestherapie

Die empfohlene Auswahl an anti-hyperglykämischen Präparaten entspricht jener für jüngere Patienten und orientiert sich an Komorbiditäten, Verträglichkeit, Nebenwirkungen und Kontraindikationen. Einmal täglich zu verabreichende Präparate sowie sinnvolle Kombinationspräparate sind Adhärenz-fördernd und erhöhen somit die Therapieverlässlichkeit [72].

Bei Metformin sind allfällige Kontraindikationen aufgrund reduzierter Organfunktionen zu beachten (Niere, Leber, Herz). Metformin darf bei Patienten mit eingeschränkter, aber stabiler Nierenfunktion bis zu einer geschätzten GFR von 30 ml/min verwendet werden. Bei einer GRF von < 30 ml/min ist Metformin kontraindiziert. Bei einer GFR von 30–45 ml/min beträgt die maximale Tagesdosis 1000 mg aufgeteilt auf zwei Dosen. Eine engmaschige Kontrolle der Nierenfunktion sollte erfolgen (alle 3–6 Monate) [73, 74]. Ein generelles Alterslimit besteht aber nicht. Metformin eignet sich nicht zur Behandlung untergewichtiger Patienten. Da es unter Langzeittherapie mit Metformin zu einem Vitamin B12 Mangel kommen kann, wird empfohlen, den Vitamin B12 Spiegel in regelmäßigen Abständen zu kontrollieren, vor allem wenn eine Anämie oder eine periphere Neuropathie vorliegt. [75–79].

SGLT-2-Hemmer wirken durch eine Hemmung des Natrium-Glukose-Cotransporters 2 im proximalen Tubulus des Nephrons mit nachfolgender verminderter Resorption von Glukose und vermehrter Ausscheidung über den Harn über einen insulinunabhängigen Mechanismus und führen dadurch zu keiner Hypoglykämie in der Monotherapie [80]. In Studien zur kardiovaskulären Sicherheit konnte der primäre kombinierte kardiovaskuläre Endpunkt (3-MACE) durch die SGLT‑2 Hemmer Empa- und Canagliflozin und der primäre kombinierte Endpunkt aus kardiovaskulärem Tod und Hospitalisierungen aufgrund von Herzinsuffizienz durch Dapagliflozin signifikant gesenkt werden. Auch die ältere Subgruppe (≥ 65 Jahre) profitierte in ähnlichem Ausmaß verglichen mit < 65 Jährigen [81–84]. Zusätzlich konnte bei Patienten mit chronischer Niereninsuffizienz eine Verlangsamung der Progression und bei Herzinsuffizienz ein Benefit gezeigt werden [85–89]. Bei Vorliegen einer chronischen Niereninsuffizienz oder Vorliegen einer Herzinsuffizienz wird daher auch beim älteren Patienten der Einsatz der SGLT‑2 Hemmer unter Abwägung von Nutzen und Risiko zur Organprotektion empfohlen (siehe auch Leitlinie Antihyperglykämische Therapie bei Diabetes mellitus Typ 2).

Aufgrund möglicher Nebenwirkungen, wie urogenitaler Infekte oder einem vermehrten Volumenverlust [90–96], aber auch unter laufender Diuretikagabe, sollte der Einsatz der SGLT-2-Hemmer bei älteren Patienten mit Vorsicht erfolgen. Vor größeren Operationen müssen SGLT-2-Inhibitoren wegen des Risikos der Begünstigung der Entwicklung einer euglykämischen Ketoazidose pausiert werden (siehe dazu auch das Kapitel Perioperatives Diabetesmanagement). Generell gelten SGLT-2-Inhibitoren als „Sick-Days-Off-Drugs“ und sind daher bei schweren Krankheitszuständen (z. B. fieberhaften Infekten) und bei längeren Episoden der Nahrungskarenz ebenfalls zu pausieren.

GLP-1-Analoga müssen in der Regel subkutan verabreicht werden, wodurch ein ausreichender Visus sowie motorische und kognitive Fertigkeiten vorausgesetzt werden müssen. Sie führen über eine Verzögerung der Magenentleerung und Verstärkung des Sättigungsgefühls im ZNS zu einer Gewichtsreduktion [97], welche bei älteren Menschen nicht immer erwünscht ist. In einer Reihe von klinischen Studien wurde ein kardiovaskulärer Nutzen gezeigt [98] (siehe auch Leitlinie Medikamentöse Therapie des Typ 2 Diabetes mellitus). Der Einsatz sollte somit individuell abgewogen werden und ist bei Untergewicht, ungewolltem Gewichtsverlust oder Kachexie zu vermeiden.

DPP-4-Hemmer sind für ältere Patienten prinzipiell eine gut verträgliche Medikamentengruppe und verursachen in der Monotherapie kein erhöhtes Hypoglykämierisiko. Bei Sitagliptin, Saxagliptin, Vildagliptin und Alogliptin [99–103] muss ab einer GFR < 50 ml/min die Dosis reduziert werden, da sie zu einem Großteil renal ausgeschieden werden. Eine Anwendung bei dialysepflichtiger Niereninsuffizienz (GFR < 15 ml/min) wird nicht empfohlen. Linagliptin hingegen wird größtenteils unverstoffwechselt über die Galle und den Darm ausgeschieden, und muss bei der Gabe nicht an die Leber- und Nierenfunktion angepasst werden [104, 105].

Bezüglich Glitazonen ist in erster Linie auf Herzinsuffizienz (NYHA 1–4) sowie auf Ödemneigung als Kontraindikation für deren Einsatz beim älteren Patienten zu achten. Weiters kann es zu einer erhöhten Frakturrate vor allem bei postmenopausalen Frauen kommen [106], weshalb eine Gabe bei bereits bekannter Osteoporose nur kritisch erfolgen sollte. Ein erhöhtes Hypoglykämierisiko liegt in der Monotherapie nicht vor. Der Einsatz der Glitazone sollte beim geriatrischen Patienten aufgrund des Nebenwirkungsprofiles stets nach individueller Abwägung erfolgen.

Sulfonylharnstoffe und Glinide sollten aufgrund des Hypoglykämierisiko bei geriatrischen Patienten nur mit Vorsicht eingesetzt werden. Da die Rate an schweren Hypoglykämien bei fortgeschrittener Niereninsuffizienz zunimmt, sollte der Einsatz von Sulfonylharnstoffen generell ab einer GFR < 30 ml/min vermieden werden.

## Insulintherapie

Aufgrund oben angeführter Limitierungen der derzeit verfügbaren oralen Antidiabetika sowie eines klinisch relevanten Insulinmangels ist bei betagten Personen mit Diabetes mellitus häufig der Beginn einer Insulintherapie geboten, vor allem dann, wenn eine chronische Glukosurie sowie ein ungewollter Gewichtsverlust auftreten. Die Insulintherapie sollte individuell auf die Bedürfnisse und Möglichkeiten des Patienten und seines sozialen Umfeldes abgestimmt werden. In anderen Leitlinien wurde die Austestung der kognitiven und motorischen Fähigkeiten mittels Durchführung des Uhrentestes [12] oder Geldzähl-Tests [28] empfohlen, um zu erkennen, welche Patienten bei der Einschulung auf eine Insulintherapie Schwierigkeiten haben könnten. Ein Screening auf kognitive Beeinträchtigung sollte durchgeführt werden, wenn z. B. Fehler bei der Umsetzung der Insulintherapie, Schwierigkeiten bei der Berechnung der Kohlenhydratmenge auftreten, Mahlzeiten ausgelassen werden oder vermehrt Hypoglykämien auftreten [43]. Meist empfiehlt sich ein möglichst einfaches und weitgehend sicheres Therapieregime. Entscheidende Faktoren für eine erfolgreiche und sichere Insulintherapie im Alter sind vor allem alterstaugliche Insulinspritzgeräte (gute Ablesbarkeit durch große Displays; einfache und möglichst fehlerfreie Bedienbarkeit; bei Bedarf vorgefüllte „Fertigspritzen zum Einmalgebrauch“ mit fixer Vordosierungsoption). Ebenfalls erforderlich sind alters- bzw. blindentaugliche Blutzuckerselbstmessgeräte.

## Diabetesschulung im Alter

Schulungsinhalte und -präsentationen müssen altersgerecht sein [107]:

- *Inhalte*: Hypoglykämie; Insulinspritzen; Selbstmessung; Ernährung; Füße;
- *Präsentation*: kompakte Botschaften, Praxisnähe, kurze Lektionen, häufige Wiederholungen, Kleingruppe oder Einzelschulung;
- Einbeziehung Angehöriger und des sozialen Umfelds.

Durch eine strukturierte geriatrische Schulung kann eine signifikante Verbesserung des HbA1c und eine deutliche Senkung der Häufigkeit von symptomatischen Hypoglykämien (ohne Fremdhilfe) um ungefähr 50 % erreicht werden [107].

## Insulintherapie bei Typ 2 Diabetes

Eine Insulintherapie wird bei älteren Patienten mit Typ 2 Diabetes sowohl von ärztlicher als auch von Patientenseite oft zu zurückhaltend eingesetzt, aus Angst, sie sei zu gefährlich oder zu kompliziert. Dabei kann – unter Berücksichtigung angepasster Zielwerte – eine Insulintherapie bei entsprechender Indikationsstellung helfen, Therapieziele zu erreichen und die Zahl der täglichen Tabletten zu reduzieren. Heute sind langwirksame Insulinanaloga mit flachem Wirkprofil verfügbar (s. Leitlinie Insulintherapie), die relativ einfach als Monotherapie oder zusätzlich zu einer oralen Therapie auch bei älteren Menschen eingesetzt werden können. Die Lebensqualität kann durch den anabolen Effekt einer Insulintherapie oft deutlich verbessert werden. Viele betagte Menschen führen eine durch Hyperglykämie bedingte Müdigkeit oft fälschlicherweise auf das Alter zurück. Vor dem Beginn mit einer Insulintherapie muss geprüft werden, ob der ältere Patient physisch und kognitiv in der Lage ist, den Insulin Pen zu bedienen und die Blutglukose selbst zu kontrollieren. Gegebenenfalls muss dafür eine Unterstützung organisiert werden. Auch die Fähigkeit, Hypoglykämien rechtzeitig wahrnehmen zu können, ist von großer Bedeutung. Die Hypoglykämiewahrnehmung ist bei älteren Menschen reduziert und die gegenregulatorischen Mechanismen sind im Alter weniger wirksam als bei jüngeren Patienten [59]. Generell soll Insulin bei älteren Menschen sehr vorsichtig titriert werden, um Hypoglykämien zu vermeiden. Begonnen werden kann zum Beispiel mit einer Morgendosis eines langwirksamen Insulins oder mit einer Abenddosis eines intermediär lang wirksamen Insulins in einer Dosierung von 8 Einheiten oder 0,1 Einheit/kg Körpergewicht, in manchen Fällen auch niedriger. Von Vorteil ist bei den langwirksamen Analoginsulinen die Möglichkeit der zeitlich flexiblen Applikation. Die Dosierung kann dann zum Beispiel einmal wöchentlich mittels eines einfachen Algorithmus angepasst werden. Bei schlechter Nierenfunktion ist der Katabolismus von Insulin verlangsamt; ab einer GFR < 50 ml/min wird weniger Insulin benötigt.

Es sollte die Insulintherapie möglichst einfach gehalten werden, z. B. mit einer basal unterstützten oralen Therapie (BOT). Ist eine Basalinsulintherapie aber nicht ausreichend, so sollte zunächst die Gabe von GLP‑1 Rezeptoragonisten in Erwägung gezogen, falls sie noch nicht Teil des Therapieregimes sind. Bei Nichterreichen des Therapiezieles kann die Insulintherapie je nach individueller Situation auf eine Basis-Bolus Therapie erweitert oder auf eine Mischinsulintherapie umgestellt werden. Bei der Mischinsulintherapie sind Präparate mit einem niedrigen Anteil an rasch wirksamem Insulin (30/70, 25/75) zu bevorzugen, da im geriatrischen Setting Mahlzeiten oft nicht vollständig eingenommen werden. Bei der Basis-Bolus Therapie wird mit einer 1xtäglichen Gabe eines prandialen Insulins zur größten Mahlzeit des Tages begonnen. Wenn das HbA1c Ziel nicht erreicht wird, erfolgt eine Erweiterung auf eine 2 × oder 3 × tägliche Gabe eines prandialen Insulins.

## Typ 1 Diabetes und LADA

In der Regel besteht ein Typ 1 Diabetes oder LADA bei alten Menschen seit vielen Jahren. Das Ziel der Glukoseeinstellung muss an Allgemeinzustand, Lebenserwartung, Hypoglykämieneigung und Grad der Selbständigkeit angepasst werden. Wichtig ist es, allfällige Defizite im Selbstmanagement des Diabetes rechtzeitig zu erkennen und gegebenenfalls zu komplex gewordene Insulinschemata zu vereinfachen. Zumindest jährlich sollte deshalb auf kognitive bzw. funktionelle Störungen bei älteren Patienten mit Typ 1 Diabetes geachtet werden. Eine besondere Herausforderung ist die Therapie dementer Patienten mit Typ 1 Diabetes, die oft unkontrolliert Nahrung zu sich nehmen. Wie auch bei Typ 2 Diabetes sollten Hypoglykämien dringend vermieden werden.

## Deeskalation und Vereinfachung der Therapie

**Figure 1:**
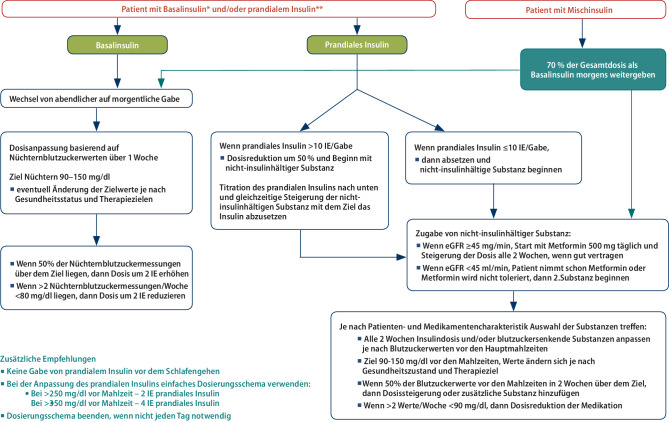

Individuell festgelegte Therapieziele sollten regelmäßig reevaluiert und angepasst werden, basierend auf chronischer Begleiterkrankungen, der kognitiven Funktion und dem funktionellen Status [43]. Daraus können sich Reduktionen der Dosierung, Umstellungen und ein Absetzen bei anti-diabetischen Medikamenten ergeben. Ziel ist es eine Überbehandlung zu vermeiden und eine Polypharmazie zu reduzieren. Wenn eine Insulintherapie durch die Patienten nicht mehr adäquat umsetzbar ist, muss eine Anpassung auf die Fähigkeiten und das soziale Umfeld der Patienten erfolgen. Die Reduktion der Therapieintensität kann sicher und auch ohne Verschlechterung der Betreuung für ältere Patienten umgesetzt werden [108], führt zu einer Reduktion von Hypoglykämien und nicht zu einer Verschlechterung der glykämischen Kontrolle. In der Abb. 1 sind Beispiele und Vorschläge für eine Vereinfachung der Insulintherapie angeführt [43].

## Die Leitlinie „Geriatrie“ der ÖDG

ist als Ergänzung zu den bestehenden Leitlinien der ÖDG mit Berücksichtigung der Besonderheiten älterer und multimorbider Menschen mit Diabetes mellitus zu sehen. Sie ist konform mit rezenten nationalen und internationalen Empfehlungen zu „Diabetes im Alter“ [12, 28, 43, 81, 109, 110].

## Interessenkonflikt

J. Huber hat von folgenden Unternehmen, die auch fördernde Mitglieder der ÖDG sind, Fortbildungsunterstützung und/oder Honorare für Vorträge und Beratung erhalten: Eli Lilly, Novartis, Novo Nordisk, Boehringer Ingelheim, Astra Zeneca, Sanofi-Aventis, Merck Sharp & Dohme, Daiichi-Sankyo, Viatris. Zusätzlich gibt J. Huber an, dass für ihn persönlich kein Interessenkonflikt vorliegt. M. Smeikal hat von folgenden Unternehmen, die auch fördernde Mitglieder der ÖDG sind, Fortbildungsunterstützung und/oder Honorare für Vorträge erhalten: Abbott, Eli Lilly, Novartis, Pfizer, Servier, Amgen, Astra Zeneca, Sanofi-Aventis. Zusätzlich gibt M. Smeikal an, dass für ihn persönlich kein Interessenkonflikt vorliegt. C. H. Saely hat von folgenden Firmen Honorare für Vorträge, Beratungstätigkeit und Forschungsunterstützung erhalten: Amgen, Astra Zeneca, Boehringer-Ingelheim, Eli-Lilly, Genericon, Genzyme, Merck, MSD, Novo Nordisk, Pfizer, Sanofi. H. Stingl hat von folgenden Unternehmen, Unterstützungen für Fortbildungen, Forschungsarbeit und/oder Honorare für Vorträge oder Beratung erhalten: Eli Lilly, Novartis, Novo Nordisk, Boehringer Ingelheim, Astra Zeneca, Sanofi-Aventis, Roche, MSD, Amgen, Abbott. Zusätzlich gibt H. Stingl an, dass für ihn persönlich kein Interessenkonflikt vorliegt. M. Lechleitner hat von folgenden Unternehmen, die auch fördernde Mitglieder der ÖDG sind, Honorare erhalten: Boehringer Ingelheim, Merck Sharp & Dome, Novartis, Novo Nordisk, Pfizer, Sanofi-Aventis. P. Fasching hat von folgenden Unternehmen, die auch fördernde Mitglieder der ÖDG sind, Forschungsunterstützungen und/oder Honorare erhalten: Abbott, AstraZeneca, Bayer Health Care, Boehringer Ingelheim, Bristol-Myers Squibb, Daiichi-Sankyo, Germania Pharmazeutika, GlaxoSmithKline Pharma, Eli Lilly, Merck Serono, Merck Sharp & Dohme, Novartis, Novo Nordisk, Pfizer, Roche, Sanofi-Aventis, Servier, Takeda. Zusätzlich gibt P. Fasching an, dass für ihn persönlich kein Interessenkonflikt vorliegt. Er legt offen, dass er in diesem Zeitraum von den genannten Firmen Honorare für Vorträge und Consulting erhalten bzw. Fortbildungsunterstützung im Rahmen der (dienst)rechtlichen Rahmenbedingungen bezogen hat (Einladung zu Kongressen) bzw. klinische Studien als PI mit einzelnen Firmen durchgeführt hat bzw. durchführt.

## References

[1] Rathmann W, Haastert B, Icks A, Löwel H, Meisinger C, Holle R (2003). High prevalence of undiagnosed diabetes mellitus in southern Germany: target populations for efficient screening. The KORA survey 2000. Diabetologia.

[2] The DECODE study group (2003). Age- and sex-specific prevalences of diabetes and impaired glucose regulation in 13 European cohorts. Diabetes Care.

[3] American Diabetes Association Professional Practice Committee (2018). Classification and diagnosis of diabetes: Standards of medical care in Diabetesd2018. Diabetes Care.

[4] Roden M (2016). Diabetes mellitus – Definition, Klassifikation und Diagnose. Wien. Klin. Wochenschr..

[5] Resnick HE, Harris MI, Brock DB, Harris TB (2000). American Diabetes Association diabetes diagnostic criteria, advancing age, and cardiovascular disease risk profiles: results from the Third National Health and Nutrition Examination Survey. Diabetes Care.

[6] Bethel MA, Sloan FA, Belsky D, Feinglos MN (2007). Longitudinal incidence and prevalence of adverse outcomes of diabetes mellitus in elderly patients. Arch Intern Med.

[7] Rosenthal MJ, Fajardo M, Gilmore S, Morley JE, Naliboff BD (1998). Hospitalization and mortality of diabetes in older adults: a 3-year prospective study. Diabetes Care.

[8] Tancredi M, Rosengren A, Svensson A-M, Kosiborod M, Pivodic A, Gudbjörnsdottir S (2015). Excess mortality among persons with type 2 diabetes. N Engl J Med.

[9] Knowler WC, Barrett-Connor E, Fowler SE, Hamman RF, Lachin JM, Walker EA, Diabetes Prevention Program Research Group, et al. Diabetes Prevention Program Research Group (2002). Reduction in the incidence of type 2 diabetes with lifestyle intervention metformin. N Engl J Med.

[10] Jefferis BJ, Lennon L, Whincup PH, Wannamethee SG (2012). Longitudinal associations between changes in physical activity and onset of type 2 diabetes in older British men: the influence of adiposity. Diabetes Care.

[11] Villareal DT, Chode S, Parimi N, Sinacore DR, Hilton T, Armamento-Villareal R (2011). Weight loss, exercise, or both and physical function in obese older adults. N Engl J Med.

[12] Meneilly GS, Knip A, Miller DB, Sherifali D, Tessier D, Zahedi A, Diabetes Canada Clinical Practice Guidelines Expert Committee (2018). Diabetes in older people. Can J Diabetes.

[13] Dorner B, Friedrich EK, Posthauer ME, American Dietetic Association (2010). Position of the American Dietetic Association: individualized nutrition approaches for older adults in health care communities. J Am Diet Assoc.

[14] Volkert D, Bauer J, Frühwald T, Gehrke I, Lechleitner M, Lenzen-Großimlinghaus R (2013). Leitlinie der Deutschen Gesellschaft für Ernährungsmedizin (DGEM) in Zusammenarbeit mit der GESKES, der AKE und der DGG. Aktuel Ernahrungsmed.

[15] Evans WJ (2004). Protein nutrition, exercise and aging. J Am Coll Nutr.

[16] Kalyani RR, Tian J, Xue QL, Walston J, Cappola AR, Fried LP (2012). Hyperglycemia and incidence of frailty and lower extremity mobility limitations in older women. J Am Geriatr Soc.

[17] Bandeen-Roche K, Seplaki CL, Huang J, Buta B, Kalyani RR, Varadhan R (2015). Frailty in older adults: a nationally representative profile in the United States. J. Gerontol. A. Biol. Sci. Med. Sci..

[18] Bertoni AG, Krop JS, Anderson GF, Brancati FL (2002). Diabetes-related morbidity and mortality in a national sample of U.S. elders. Diabetes Care.

[19] Gregg EW, Beckles GLA, Williamson DF, Leveille SG, Langlois JA, Engelgau MM (2000). Diabetes and physical disability among older U.S. adults. Diabetes Care.

[20] Krop JS, Powe NR, Weller WE, Shaffer TJ, Saudek CD, Anderson GF (1998). Patterns of expenditures and use of services among older adults with diabetes: implications for the transition to capitated managed care. Diabetes Care.

[21] Fried LP, Tangen CM, Walston J, Newman AB, Hirsch C, Gottdiener J (2001). Frailty in older adults: evidence for a phenotype. J. Gerontol. A. Biol. Sci. Med. Sci..

[22] Sinclair A, Morley J (2013). Frailty and diabetes. Lancet.

[23] Cruz-Jentoft A, Baeyens J, Bauer J, Boirie Y, Cederholm T, Landi F (2010). Sarcopenia: European consensus on definition and diagnosis: report of the European working group on sarcopenia in older people. Age Ageing.

[24] Leenders M, Verdijk LB, van der Hoeven L, Adam JJ, van Kranenburg J, Nilwik R (2013). Patients with type 2 diabetes show a greater decline in muscle mass, muscle strength, and functional capacity with aging. J Am Med Dir Assoc.

[25] Volpato S, Bianchi L, Lauretani F, Lauretani F, Bandinelli S, Guralnik JM (2012). Role of muscle mass and muscle quality in the association between diabetes and gait speed. Diabetes Care.

[26] Woo J, Leung J, Morley JE (2014). Validating the SARC-F: a suitable community screening tool for sarcopenia?. J Am Med Dir Assoc.

[27] Morley JE, Vellas B, Abellan van Kan G, Anker SD, Bauer JM, Bernabei R (2013). Frailty consensus: a call to action. J Am Med Dir Assoc.

[28] Bahrmann A, Bahrmann P, Baumann J, Bauer J, Brückel E, Dreyer M (2018). S2k-Leitlinie Diagnostik, Therapie und Verlaufskontrolle des Diabetes mellitus im Alter: 2. Auflage 2018 – AWMF-Register-Nr. 057-017. Diabetol Stoffwechsel.

[29] Pariser G, Hager K, Gillette P, Golemboski K, Jackson K (2014). Active steps for diabetes: a community-campus partnership addressing frailty and diabetes. Diabetes Educ.

[30] Gordon PL, Vannier E, Hamada K, Layne J, Hurley BF, Roubenoff R (2006). Resistance training alters cytokine gene expression in skeletal muscle of adults with type 2 diabetes. Int J Immunopathol Pharmacol.

[31] Reusch JEB, Bridenstine M, Regensteiner JG (2013). Type 2 diabetes mellitus and exercise impairment. Rev Endocr Metab Disord.

[32] Bauer J, Biolo G, Cederholm T, Cesari M, Cruz-Jentoft AJ, Morley JE (2013). Evidence-based recommendations for optimal dietary protein intake in older people: a position paper from the prot-age study group. J Am Med Dir Assoc.

[33] Rawlings AM, Sharrett AR, Schneider ALC, Coresh J, Albert M, Couper D (2014). Diabetes in midlife and cognitive change over 20 years: a cohort study. Ann Intern Med.

[34] Li W, Huang E (2016). An update on type 2 diabetes mellitus as a risk factor for dementia. J. Alzheimers Dis..

[35] Yaffe K, Falvey C, Hamilton N, Schwartz AV, Simonsick EM, Satterfield S (2012). Diabetes, glucose control, and 9-year cognitive decline among older adults without dementia. Arch Neurol.

[36] Zheng B, Su B, Price G, Tzoulaki I, Ahmadi-Abhari S, Middleton L. Glycemic control, diabetic complications, and risk of dementia in patients with diabetes: results from a large U.K. cohort study. 2021. https://diabetesjournals.org/care/article/44/7/1556/138822/Glycemic-Control-Diabetic-Complications-and-Risk. Zugegriffen: 7. Juli 2022, Diabetes Care [Internet]. 10.2337/dc20-2850 34035076

[37] van Gennip ACE, Stehouwer CDA, van Boxtel MPJ, Verhey FRJ, Koster A, Kroon AA, et al. Association of type 2 diabetes, according to the number of risk factors within target range, with structural brain abnormalities, cognitive performance, and risk of dementia. 2021. https://diabetesjournals.org/care/article/44/11/2493/138517/Association-of-Type-2-Diabetes-According-to-the. Zugegriffen: 7. Juli 2022, Diabetes Care [Internet]. 10.2337/dc21-0149 PMC9612883 34588209

[38] Kessler RC, Berglund P, Demler O, Jin R, Koretz D, Merikangas KR (2003). The epidemiology of major depressive disorder: results from the National Comorbidity Survey Replication (NCS-R). J Am Med Assoc.

[39] Katon W, Sondergaard Pedersen H, Riisgaard Ribe A, Fenger-Grøn M, Davydow D, Boch Waldorff F, Vestergaard M (2015). Effect of depression and diabetes mellitus on the risk for dementia: a national population-based cohort study. Jama Psychiatry.

[40] van Dooren FEP, Nefs G, Schram MT, Verhey FRJ, Denollet J, Pouwer F (2013). Depression and risk of mortality in people with diabetes mellitus: a systematic review and meta-analysis. PLoS ONE.

[41] Black SA, Markides KS, Ray LA (2003). Depression predicts increased incidence of adverse health outcomes in older Mexican Americans with type 2 diabetes. Diabetes Care.

[42] Kimbro LB, Mangione CM, Steers WN, Duru OK, McEwen L, Karter A (2014). Depression and all-cause mortality in persons with diabetes mellitus: Are older adults at higher risk? Results from the translating research into action for diabetes study. J Am Geriatr Soc.

[43] American Diabetes Association Professional Practice Committee. Older adults: standards of medical care in diabetes—2022. 2022. https://diabetesjournals.org/care/article/45/Supplement_1/S195/138920/13-Older-Adults-Standards-of-Medical-Care-in. Zugegriffen: 7. Juli 2022, Diabetes Care [Internet].

[44] Folstein MF, Folstein SE, McHugh PR. “Mini-mental state”. A practical method for grading the cognitive state of patients for the clinician. 1975. https://jhu.pure.elsevier.com/en/publications/mini-mental-state-a-practical-method-for-grading-the-cognitive-st-6. Zugegriffen: 7. Juli 2022. 10.1016/0022-3956(75)90026-6 1202204

[45] Borson S, Scanlan JM, Chen P, Ganguli M (2003). The mini-cog as a screen for dementia: validation in a population-based sample. J. Am. Geriatr. Soc..

[46] Nasreddine ZS, Phillips NA, Bédirian V, Charbonneau S, Whitehead V, Collin I (2005). The Montreal Cognitive Assessment, MoCA: A brief screening tool for mild cognitive impairment. J Am Geriatr Soc.

[47] APA task force on the evaluation of dementia and age-related cognitive change. Guidelines for the evaluation of dementia and age-related cognitive change. 2021. https://www.apa.org/practice/guidelines/. Zugegriffen: 8. Juli 2022, Approved by APA council of representatives February 2021.

[48] Huang ES, Liu JY, Moffet HH, John PM, Karter AJ (2011). Glycemic control, complications, and death in older diabetic patients: the diabetes and aging study. Diabetes Care.

[49] Gerstein HC, Miller ME, Byington RP, Goff DC, Bigger JT, Buse JB (2008). Effects of intensive glucose lowering in type 2 diabetes. (ACCORD study results). N Engl J Med.

[50] Tan HH, McAlpine RR, James P, Thompson P, McMurdo MET, Morris AD (2004). Diagnosis of type 2 diabetes at an older age: effect on mortality in men and women. Diabetes Care.

[51] Landgraf R, Kellerer M, Fach E, Gallwitz B, Hamann A, Joost HG (2013). Praxisempfehlungen DDG/DGIM: Therapie des Typ-2-Diabetes. Diabetol Stoffwechsel.

[52] James PA, Oparil S, Carter BL, Cushman WC, Dennison-Himmelfarb C, Handler J (2014). 2014 evidence-based guideline for the management of high blood pressure in adults. JAMA.

[53] Sue Kirkman M, Briscoe VJ, Clark N, Florez H, Haas LB, Halter JB (2012). Diabetes in older adults: a consensus report. J Am Geriatr Soc.

[54] American Diabetes Association (2018). Diabetes care: standards of medical care in diabetes—2018. Diabetes Care.

[55] Williams B, Mancia G, Spiering W, Rosei EA, Azizi M, Burnier M (2018). 2018 ESC/ESH guidelines for themanagement of arterial hypertension. Eur Heart J.

[56] Mach F, Baigent C, et al. 2019 ESC/EAS guidelines for the management of dyslipidaemias: lipid modification to reduce cardiovascular risk. 2020. https://academic.oup.com/eurheartj/article-abstract/41/1/111/5556353. Zugegriffen: 8. Juli 2022, The Task Force for the management of. academic.oup.com [Internet].

[57] Visseren FLJ, Mac HF, Smulders YM, Carballo D, Koskinas KC, Bäck M (2021). ESC guidelines on cardiovascular disease prevention in clinical practice. Eur Heart J.

[58] Abbatecola AM, Rizzo MR, Barbieri M, Grella R, Arciello A, Laieta MT (2006). Postprandial plasma glucose excursions and cognitive functioning in aged type 2 diabetics. Neurology.

[59] Bremer JP, Jauch-Chara K, Hallschmid M, Schmid S, Schultes B (2009). Hypoglycemia unawareness in older compared with middle-aged patients with type 2 diabetes. Diabetes Care.

[60] Whitmer RA, Karter AJ, Yaffe K, Quesenberry CP, Selby JV (2009). Hypoglycemic episodes and risk of dementia in older patients with type 2 diabetes mellitus. JAMA.

[61] Punthakee Z, Miller ME, Launer LJ, Williamson JD, Lazar RM, Cukierman-Yaffee T (2012). Poor cognitive function and risk of severe hypoglycemia in type 2 diabetes: post hoc epidemiologic analysis of the ACCORD trial. Diabetes Care.

[62] Chobanian AV, Bakris GL, Black HR, Cushman WC, Green LA, Izzo JL (2003). The seventh report of the joint national committee on prevention, detection, evaluation, and treatment of high blood pressure: the JNC 7 report. JAMA.

[63] Beckett NS, Peters R, Fletcher AE, Staessen JA, Liu L, Dumitrascu D (2008). Treatment of hypertension in patients 80 years of age and older. N Engl J Med.

[64] Wright JT, Williamson JD, Whelton PSK, Snyder JK, Sink KM, Rocco MV, SPRINT Research Group (2015). A randomized trial of intensive versus standard blood-pressure control. N Engl J Med.

[65] Rahimi K, Bidel Z, Nazarzadeh M, Copland E, Canoy D, Wamil M (2021). Age-stratified and blood-pressure-stratified effects of blood-pressure-lowering pharmacotherapy for the prevention of cardiovascular disease and death: an individual participant-level data meta-analysis. Lancet.

[66] Schäfer HH, De Villiers JN, Sudano I, Dischinger S, Theus GR, Zilla P (2012). Recommendations for the treatment of hypertension in the elderly and very elderly—a scotoma within international guidelines. Swiss Med Wkly.

[67] Catapano AL, Graham I, De Backer G, Wiklund O, Chapman MJ, Drexel H (2016). 2016 ESC/EAS guidelines for the management of dyslipidaemias. Eur Heart J.

[68] Gencer B, Marston NA, Im KA, Cannon CP, Sever P, Keech A, et al. Efficacy and safety of lowering LDL cholesterol in older patients: a systematic review and meta-analysis of randomised controlled trials. 2020. http://www.thelancet.com/article/S0140673620323321/fulltext. Zugegriffen: 7. Juli 2022, Lancet [Internet]. 10.1016/S0140-6736(20)32332-1 PMC8015314 33186535

[69] Collins R, Armitage J, Parish S, Sleight P, Peto R (2002). MRC/BHF heart protection study of cholesterol lowering with simvastatin in 20 536 high-risk individuals: a randomised placebo-controlled trial. Lancet.

[70] Shepherd J, Blauw GJ, Murphy MB, Bollen ELEM, Buckley BM, Cobbe SM (2002). Pravastatin in elderly individuals at risk of vascular disease (PROSPER): a randomised controlled trial. Lancet.

[71] Forman D, Wenger NK (2013). What do the recent American Heart Association/American College of Cardiology Foundation clinical practice guidelines tell us about the evolving management of coronary heart disease in older adults?. J Geriatr Cardiol.

[72] Donnan PT, MacDonald TM, Morris AD (2002). Adherence to prescribed oral hypoglycaemic medication in a population of patients with type 2 diabetes: a retrospective cohort study. Diabet Med.

[73] Lipska KJ, Bailey CJ, Inzucchi SE (2011). Use of metformin in the setting of mild-to-moderate renal insufficiency. Diabetes Care.

[74] Inzucchi SE, Bergenstal RM, Buse JB, Diamant M, Ferrannini E, Nauck M (2015). Management of hyperglycaemia in type 2 diabetes, 2015: a patient-centred approach. Diabetologia.

[75] Kos E, Liszek M, Emanuele M, Durazo-Arvizu R, Camacho P (2012). Effect of Metformin therapy on vitamin D and vitamin B_12_ levels in patients with type 2 diabetes mellitus. Endocr Pract.

[76] De Jager J, Kooy A, Lehert P, Wulffelé MG, Van Der Kolk J, Bets D (2010). Long term treatment with metformin in patients with type 2 diabetes and risk of vitamin B-12 deficiency: randomised placebo controlled trial. BMJ.

[77] Ting RZW, Szeto CC, Chan MHM, Ma KK, Chow KM (2006). Risk factors of vitamin B12 deficiency in patients receiving metformin. Arch Intern Med.

[78] Aroda VR, Edelstein SL, Goldberg RB, Knowler WC, Marcovina SM, Orchard TJ (2016). Long-term metformin use and vitamin B12 deficiency in the diabetes prevention program outcomes study. J Clin Endocrinol Metab.

[79] Out M, Kooy A, Lehert P, Schalkwijk CA, Stehouwer CDA (2018). Long-term treatment with metformin in type 2 diabetes and methylmalonic acid: Post hoc analysis of a randomized controlled 4.3 year trial. J Diabetes Complications.

[80] Ferrannini E, Solini A (2012). SGLT2 inhibition in diabetes mellitus: rationale and clinical prospects. Nat Rev Endocrinol.

[81] Davies MJ, D’Alessio DA, Fradkin J, Kernan WN, Mathieu C, Mingrone G (2018). Management of hyperglycaemia in type 2 diabetes, 2018. A consensus report by the American Diabetes Association (ADA) and the European Association for the Study of Diabetes (EASD). Diabetologia.

[82] Zinman B, Wanner C, Lachin JM, Fitchett D, Bluhmki E, Hantel S (2015). Empagliflozin, cardiovascular outcomes, and mortality in type 2 diabetes. N Engl J Med.

[83] Neal B, Perkovic V, Matthews DR (2017). Canagliflozin and cardiovascular and renal events in type 2 diabetes. N Engl J Med.

[84] Wiviott SD, Raz I, Bonaca MP, Mosenzon O, Kato ET, Cahn A (2019). Dapagliflozin and cardiovascular outcomes in type 2 diabetes. N Engl J Med.

[85] McMurray JJV, Solomon SD, Inzucchi SE, Køber L, Kosiborod MN, Martinez FA (2019). Dapagliflozin in patients with heart failure and reduced ejection fraction. N Engl J Med.

[86] Anker SD, Butler J, Filippatos G, Ferreira JP, Bocchi E, Böhm M (2021). Empagliflozin in heart failure with a preserved ejection fraction. N Engl J Med.

[87] Packer M, Anker SD, Butler J, Filippatos G, Pocock SJ, Carson P (2020). Cardiovascular and renal outcomes with Empagliflozin in heart failure. N Engl J Med.

[88] Heerspink HJL, Stefánsson V, Correa-Rotter R, Chertow GM, Greene T, Hou F‑F, et al. Dapagliflozin in patients with chronic kidney disease (DAPA-CKD study). 2020. http://rcpi-live-cdn.s3.amazonaws.com/wp-content/uploads/2020/11/Dr-Katie-Liston-Winner-Review-Article-Sept-to-Oct-2020.pdf. Zugegriffen: 8. Juli 2022, rcpi-live-cdn.s3.amazonaws.com [Internet].

[89] Perkovic V, Jardine MJ, Neal B, Bompoint S, Heerspink HJ, Charytan DM, et al. Canagliflozin and renal outcomes in type 2 diabetes and nephropathy (CREDENCE trial). 2019. http://rcpi-live-cdn.s3.amazonaws.com/wp-content/uploads/2020/04/Other-Excellent-review-Dr-Cliona-Cowhig.pdf. Zugegriffen: 8. Juli 2022, rcpi-live-cdn.s3.amazonaws.com [Internet].

[90] Monami M, Nardini C, Mannucci E (2014). Efficacy and safety of sodium glucose co-transport-2 inhibitors in type 2 diabetes: a meta-analysis of randomized clinical trials. Diabetes Obes Metab.

[91] Johnsson KM, Ptaszynska A, Schmitz B, Sugg J, Parikh SJ, List JF (2013). Vulvovaginitis and balanitis in patients with diabetes treated with dapagliflozin. J Diabetes Complicat.

[92] Johnsson KM, Ptaszynska A, Schmitz B, Sugg J, Parikh SJ, List JF (2013). Urinary tract infections in patients with diabetes treated with dapagliflozin. J Diabetes Complicat.

[93] Nyirjesy P, Sobel JD, Fung A, Mayer C, Capuano G, Ways K (2014). Genital mycotic infections with canagliflozin, a sodium glucose co-transporter 2 inhibitor, in patients with type 2 diabetes mellitus: a pooled analysis of clinical studies. Curr Med Res Opin.

[94] Cefalu WT, Leiter LA, Yoon KH, Arias P, Niskanen L, Xie J (2013). Efficacy and safety of canagliflozin versus glimepiride in patients with type 2 diabetes inadequately controlled with metformin (CANTATA-SU): 52 week results from a randomised, double-blind, phase 3 non-inferiority trial. Lancet.

[95] Schernthaner G, Gross JL, Rosenstock J, Guarisco M, Fu M, Yee J (2013). Canagliflozin compared with sitagliptin for patients with type 2 diabetes who do not have adequate glycemic control with metformin plus sulfonylurea: a 52-week randomized trial. Diabetes Care.

[96] Vasilakou D, Karagiannis T, Athanasiadou E, Mainou M, Liakos A, Bekiari E (2013). Sodium-glucose cotransporter 2 inhibitors for type 2 diabetes: a systematic review and meta-analysis. Ann Intern Med.

[97] Drucker DJ, Nauck MA (2006). The incretin system: glucagon-like peptide-1 receptor agonists and dipeptidyl peptidase-4 inhibitors in type 2 diabetes. Lancet.

[98] American Diabetes Association Professional Practice Committee. Cardiovascular disease and risk management: standards of medical care in diabetes—2022. 2021. https://diabetesjournals.org/care/article-abstract/45/Supplement_1/S144/138910. Zugegriffen: 8. Juli 2022, Am Diabetes Assoc [Internet].

[99] Stafford S, Elahi D, Meneilly GS (2011). Effect of the dipeptidyl peptidase-4 inhibitor sitagliptin in older adults with type 2 diabetes mellitus. J Am Geriatr Soc.

[100] Barzilai N, Guo H, Mahoney EM, Caporossi S, Golm GT, Langdon RB (2011). Efficacy and tolerability of sitagliptin monotherapy in elderly patients with type 2 diabetes: a randomized, double-blind, placebo-controlled trial. Curr Med Res Opin.

[101] Schwartz SL (2010). Treatment of elderly patients with type 2 diabetes mellitus: a systematic review of the benefits and risks of dipeptidyl peptidase-4 inhibitors. Am J Geriatr Pharmacother.

[102] Pratley RE, McCall T, Fleck PR, Wilson CA, Mekki Q (2009). Alogliptin use in elderly people: A pooled analysis from phase 2 and 3 studies. J Am Geriatr Soc.

[103] Defronzo RA, Fleck PR, Wilson CA, Mekki Q (2008). Efficacy and safety of the dipeptidyl peptidase-4 inhibitor alogliptin in patients with type 2 diabetes and inadequate glycemic control: a randomized, double-blind, placebo-controlled study. Diabetes Care.

[104] Barnett AH (2012). The role of GLP-1 mimetics and basal insulin analogues in type 2 diabetes mellitus: guidance from studies of liraglutide. Diabetes Obes Metab.

[105] Graefe-Mody U, Friedrich C, Port A, Ring A, Retlich S, Heise T (2011). Effect of renal impairment on the pharmacokinetics of the dipeptidyl peptidase-4 inhibitor linagliptin. Diabetes Obes Metab.

[106] Dormandy JA, Charbonnel B, Eckland DJA, Erdmann E, Massi-Benedetti M, Moules IK (2005). Secondary prevention of macrovascular events in patients with type 2 diabetes in the PROactive Study (PROspective pioglitAzone Clinical Trial In macroVascular Events): a randomised controlled trial. Lancet.

[107] Braun AK, Kubiak T, Kuntsche J, Meier-Höfig M, Müller UA, Feucht I (2009). SGS: a structured treatment and teaching programme for older patients with diabetes mellitus—a prospective randomised controlled multi-centre trial. Age Ageing.

[108] Seidu S, et al. De-intensification in older people with type 2 diabetes: why, when and for whom? 2021. https://www.thelancet.com/journals/lanhl/article/PIIS2666-7568(21)00204-X/fulltext. Zugegriffen: 8. Juli 2022, thelancet.com [Internet].

[109] Sinclair A, Morley JE, Rodriguez-Mañas L, Paolisso G, Bayer T, Zeyfang A (2012). Diabetes mellitus in older people: position statement on behalf of the International Association of Gerontology and Geriatrics (IAGG), the European Diabetes Working Party for Older People (EDWPOP), and the International Task Force of Experts in Diabetes. J Am Med Dir Assoc.

[110] American Diabetes Association (2018). Older adults: Standards of medical care in Diabetesd2018. Diabetes Care.

